# STELAR: Spatio-temporal Tensor Factorization with Latent Epidemiological Regularization

**Published:** 2020-12-08

**Authors:** Nikos Kargas, Cheng Qian, Nicholas D. Sidiropoulos, Cao Xiao, Lucas M. Glass, Jimeng Sun

**Affiliations:** 1 Dept. of ECE, University of Minnesota; 2 Analytics Center of Excellence, IQVIA; 3 Dept. of ECE, University of Virginia; 4 Dept. of Statistics, Temple University; 5 Dept. of CS, University of Illinois Urbana-Champaign

## Abstract

Accurate prediction of the transmission of epidemic diseases such as COVID-19 is crucial for implementing effective mitigation measures. In this work, we develop a tensor method to predict the evolution of epidemic trends for many regions simultaneously. We construct a 3-way spatio-temporal tensor (location, attribute, time) of case counts and propose a nonnegative tensor factorization with latent epidemiological model regularization named STELAR. Unlike standard tensor factorization methods which cannot predict slabs ahead, STELAR enables long-term prediction by incorporating latent temporal regularization through a system of discrete-time difference equations of a widely adopted epidemiological model. We use *latent* instead of location/attribute-level epidemiological dynamics to capture common epidemic profile sub-types and improve collaborative learning and prediction. We conduct experiments using both county- and state-level COVID-19 data and show that our model can identify interesting latent patterns of the epidemic. Finally, we evaluate the predictive ability of our method and show superior performance compared to the baselines, achieving up to 21% lower root mean square error and 25% lower mean absolute error for county-level prediction.

## Introduction

Pandemic diseases such as the novel coronavirus disease (COVID-19) pose a serious threat to global public health, economy, and daily life. Accurate epidemiological measurement, modeling, and tracking are needed to inform public health officials, government executives, policy makers, emergency responders, and the public at large. Two types of epidemiological modeling methods are popular today:

- **Mechanistic models** define a set of ordinary differential equations (ODEs) which capture the epidemic transmission patterns and predict the long-term trajectory of the outbreak. These models include the Susceptible-Infected-Recovered (SIR) model (Kermack and McKendrick 1927), the Susceptible-Exposed-Infected-Recovered (SEIR) (Cooke and Van Den Driessche 1996) and their variants e.g, SIS, SIRS, and delayed SIR. These models have a small number of parameters which are determined via curve fitting. These types of models do not require much training data, but they are quite restrictive and cannot leverage rich information.
- **Machine learning models** such as deep learning, model the epidemiological trends as a set of regression problems (Yang et al. 2020; Toda 2020; He, Peng, and Sun 2020; Chimmula and Zhang 2020; Tomar and Gupta 2020). These models are able to learn the trajectory of the outbreak only from data and can perform very well especially in short-term prediction. However, they usually require a large amount of training data.

This paper extends Canonical Polyadic Decomposition (CPD) (Harshman 1970) via epidemiological model (e.g., SIR and SEIR) regularization, which integrates spatio-temporal real-world case data and exploits the correlation between different regions for long-term epidemic prediction. CPD is a powerful tensor model with successful applications in many fields (Papalexakis, Faloutsos, and Sidiropoulos 2016; Sidiropoulos et al. 2017). Compared to traditional epidemiological models which cannot incorporate fine-grain observations or any kind of side information, tensors offer a natural way of representing multidimensional time evolving data and incorporate additional information (Acar, Dunlavy, and Kolda 2009; Araujo, Ribeiro, and Faloutsos 2017). CPD can capture the inherent correlations between the different modes and thanks to its uniqueness properties it can extract interpretable latent components rendering it an appealing solution for modeling and analyzing epidemic dynamics. Additionally, it can parsimoniously represent multidimensional data and can therefore learn from limited data. These two important properties differentiate CPD from neural networks and deep learning models which typically require a lot of training data and are often treated as black box models.

In this work, we propose a Spatio-temporal Tensor factorization with EpidemioLogicAl Regularization (STELAR). STELAR combines a nonnegative CPD model with a system of discrete-time difference equations to capture the epidemic transmission patterns. Unlike standard tensor factorization methods which cannot predict slabs ahead, STELAR can simultaneously forecast the evolution of the epidemic for a list of regions, and it can also perform long-term prediction.

In the experiments, we build a spatio-temporal tensor, location × attribute × time of case counts based on large real-world medical claims datasets, where the first dimension corresponds to different counties/states, the second dimension corresponds to different attributes or signals that evolve over time, such as daily new infections, deaths, number of hospitalized patients and other COVID-19 related signals, and the third is the time-window of the available signals. This spatio-temporal tensor is factorized using the proposed low-rank nonnegative tensor factorization with epidemiological model regularization STELAR. We observed that the extracted latent time components provide intuitive interpretation of different epidemic transmission patterns which traditional epidemiological models such as SIR and SEIR are lacking.

Our main contributions are summarized as follows:

- We propose STELAR, a new data-efficient tensor factorization method regularized by a disease transmission model in the latent domain to predict future slabs. We show that by jointly fitting a low-rank nonnegative CPD model with an SIR model on the time factor matrix we are able to accurately predict the evolution of epidemic trends.
- Thanks to the uniqueness properties of the CPD, our method produces interpretable prediction results. Specifically, the tensor is approximated via *K* rank-1 components, each of which is associated with a sub-type of the epidemic transmission patterns in a given list of regions. The latent time factor matrix includes *K* different patterns of the epidemic evolution and using the latent location and signal factor matrices we can identify the corresponding locations and signals associated with each pattern.
- We perform extensive experimental evaluation on both county- and state-level COVID-19 case data for 10 and 15 days-ahead prediction and show that our method outperforms standard epidemiological and machine learning models in COVID-19 pandemic prediction. Our method achieves up to 21% lower root mean square error and 25% lower mean absolute error for county-level prediction.

## Related Work

Many epidemiological and deep learning models have been applied for modeling the COVID-19 pandemic evolution. Methods based on traditional epidemic prediction models such as SIR (Kermack and McKendrick 1927) and SEIR and their variants (Cooke and Van Den Driessche 1996), rely on a system of differential equations which describe the dynamics of the pandemic (Yang et al. 2020; Toda 2020; He, Peng, and Sun 2020). These models are trained for each location independently by curve fitting.

Machine learning approaches and specifically deep learning models treat the problem as a time series prediction problem. Given as input a time series of length *L*_*w*_, *x*_*t*−*Lw*−1_, … , *x*_*t*−1_, *x*_*t*_, the goal is to output *L*_*o*_ future time points $x_{t+1},x_{t+2},\ldots ,x_{L_{o}}$. A popular and very successful method for time series prediction is the Long Short Term Memory (LSTM) network (Hochreiter and Schmidhuber 1997). Recent studies have applied LSTMs for COVID-19 prediction (Chimmula and Zhang 2020; Tomar and Gupta 2020; Yang et al. 2020). To increase the data for LSTM training, Yang et al. used the 2003 SARS-CoV data to pretrain the model before using it for COVID-19 prediction. DEFSI (Wang, Chen, and Marathe 2019) is a deep learning method based on LSTMs for influenza like illness forecasting which combines synthetic epidemic data with real data to improve epidemic forecasts.

Graph Neural Network (GNN) (Kipf and Welling 2017) is a type of neural network which operates on a graph. Kapoor et al. proposed creating a graph with spatial and temporal edges. They leverage human mobility data to improve the prediction of daily new infections (Kapoor et al. 2020). STAN (Gao et al. 2020) is an attention-based graph convolutional network which constructs edges based on geographical proximity of the different regions and regularizes the model predictions based on an epidemiological model.

Methods based on tensor factorization have been applied for various time series prediction tasks. CP Forecasting (Dunlavy, Kolda, and Acar 2011), is a tensor method which computes a low-rank CPD model and uses the temporal factor to capture periodic patterns in data. TENSORCAST (Araujo, Ribeiro, and Faloutsos 2017) is a method that forecasts time-evolving networks using coupled tensors. Both methods are not suitable for COVID-19 pandemic prediction and rely on a two-step procedure – fitting a low-rank model and then performing forecasting based on the temporal factor matrix – which as we will see later leads to performance degradation relative to our joint optimization formulation.

## Background

In this section, we review necessary background on epidemiological models and tensor decomposition that will prove useful for developing our method. Table 1 contains the notation used throughout the paper.

### Epidemiological Models

**Figure 1:**
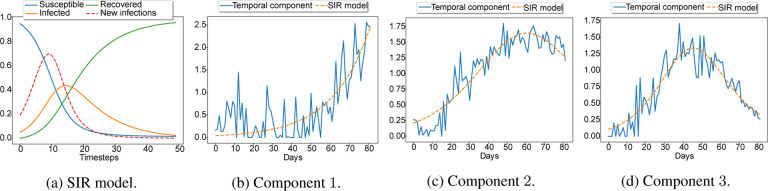
An example of an SIR model with parameters *N* = 1, *S*(0) = 0.95, *I*(0) = 0.05, *R*(0) = 0, *β* = 0.4, *γ* = 0.1 is shown in Figure 1a. The new infections curve has been scaled by 10 for visualization purposes. Figures 1b, 1c, 1d show 3 latent time components i.e., columns of the factor matrix **C** when we compute a plain nonnegative CPD of the spatio-temporal tensor.

Epidemiological models have been popular solutions for pandemic modeling. For example, The SIR model (Kermack and McKendrick 1927) is one of the most famous and paradigmatic models in mathematical epidemiology. In this model, a population is divided into susceptible, infected and recovered subpopulations. The evolution of these quantities is described by the following equations:

(1a) S(t)−S(t−1)=−βS(t−1)I(t−1)/N,

(1b) $$I(t)-I(t-1)=\frac{\beta S(t-1)I(t-1)}{N}-\gamma I(t-1),$$

(1c) R(t)−R(t−1)=γI(t−1),

where *S*(*t*), *I*(*t*) and *R*(*t*) stand for the size of susceptible, infected and recovered populations at time *t*, respectively and *N* = *S*(*t*) + *I*(*t*) + *R*(*t*) is the total population. The parameter *β* controls the rate of spread and the parameter *γ* is the recovery rate, so its inverse 1/*γ* represents the average time period that an infected individual remains infectious. In Equation (1a), the quantity *βS*(*t*)/*N* is the fraction of susceptible individuals that will contact an infected individual at time *t*. Therefore,

(2) C(t):=βS(t)I(t)/N,

denotes the new infections at time *t*. In Equation (1b), *γI*(*t*) denotes the number of individuals recovered. Therefore, the change of the infected cases at time *t* will be the difference between new infected and recovered cases as shown in Equation (1b). Given *S*(0), *I*(0) and *R*(0), we can compute *S*(*t*), *I*(*t*) and *R*(*t*) for *t* = 1, 2, … , *L*, where *L* is the size of the prediction window we are interested in. An example is shown in Figure (1a) where we have set *N* = 1, *S*(0) = 0.95, *I*(0) = 0.05, *R*(0) = 0, *β* = 0.4, *γ* = 0.1 i.e., we have normalized the different populations by the total population number *N* and set the window size to be *L* = 50.

The SEIR model is a variant of the SIR model. In this model, a population is divided into susceptible, exposed, infected and recovered subpopulations. The exposed population consists of individuals who have been infected but are not yet infectious. The SEIR model includes an additional parameter *σ* which is the rate at which the exposed individuals becoming infectious.

### Canonical Polyadic Decomposition

The Canonical Polyadic Decomposition (CPD) expresses a 3-way tensor $\underline{X}\in {\mathbb{R}}^{M\times N\times L}$ as a sum of rank-1 components, i.e.,

(3) $$\underline{X}=〚A,B,C〛=\sum_{k=1}^{K}\;a_{k}\circ b_{k}\circ c_{k},$$

where $A=\left[a_{1},\ldots ,a_{K}\right]\in {\mathbb{R}}^{M\times K}$, $B=\left[b_{1},\ldots ,b_{K}\right]\in {\mathbb{R}}^{N\times K}$, $C=\left[c_{1},\ldots ,c_{K}\right]\in {\mathbb{R}}^{L\times K}$. The rank *K* is the minimum number of components needed to synthesize $\underline{X}$. We can express the CPD of a tensor in many different ways. For example, $\underline{X}=〚A,B,C〛$ can be represented by the matrix unfolding ${\underline{X}}^{\left(1\right)}=(C⊙B)A^{T}$ where the mode-1 fibers are the rows of the resulting matrix. Using ‘role symmetry’, the mode-2 and mode-3 matrix unfoldings are given by ${\underline{X}}^{\left(2\right)}=(C⊙A)B^{T}$, ${\underline{X}}^{\left(3\right)}=(B⊙A)C^{T}$ respectively. CPD can parsimoniously represent tensors of size *M* × *N* × *L* using only (*M* + *N* + *L*) × *K* parameters. The CPD model has two very important properties that make it a very powerful tool for data analysis 1) it is universal, i.e., every tensor admits a CPD of finite rank, and 2) it is unique under mild conditions i.e., it is possible to extract the true latent factors that synthesize $\underline{X}$.

## Method

### Problem Formulation

Consider a location where we monitor *N* signals related to a pandemic over time, e.g., number of new infections, hospitalized patients, intensive care unit (ICU) patients, etc. At time *t*, the value of the *n*th signal at location *m* is denoted by *x*_*m,n,t*_. Assuming that there are *N* signals, *M* locations and *L* time points, then, the dataset can be naturally described by a 3-way spatio-temporal tensor $\underline{X}\in {\mathbb{R}}^{M\times N\times L}$, where $\underline{X}(m,n,t):=x_{m,n,t}$. Tensor $\underline{X}$ includes the evolution of all signals and regions for times 1 through *L* and we are interested in estimating the signals at time *L*+1, … , *L*+*L*_*o*_. In other words, we want to predict the frontal slabs $\underline{X}(:,:,t)$ for *L*_*o*_ timesteps ahead. However, standard tensor factorization methods cannot predict slabs ahead. It is evident from Equation 3 and the mode-3 unfolding that is impossible to impute when an entire slab $\underline{X}(:,:,t)$ is missing since we have no information regarding the corresponding row of **C**. To address this challenge, we take into consideration the transmission law dynamics of the disease. The key idea is to decompose the tensor using a CPD model and impose SIR constraints on the latent time factor.

To illustrate the key idea, we perform a preliminary experiment using a rank-5 plain nonnegative CPD on a spatio-temporal tensor $\underline{X}\in {\mathbb{R}}^{140\times 15\times 82}$ with case counts, constructed from real COVID-19 data. As shown in Figures 1b, 1c, 1d, CPD is able to extract meaningful latent components from the data. Specifically, the figures show 3 columns of the latent time factor **C** where an SIR model has been fitted after obtaining the decomposition. We observe that each columns depicts a curve similar to the one in Figure 1a i.e., the CPD can unveil the principal patterns of the epidemic evolution, and each pattern corresponds to a different pandemic phase.

Therefore, we propose solving the following constrained nonnegative CPD problem

(4) $$\underset{A,B,C,\beta ,\gamma ,s,i}{\text{min}}\;{\left\|\underline{X}-〚A,B,C〛\right\|}_{F}^{2}+\mu \left({\left\|A\right\|}_{F}^{2}+{\left\|B\right\|}_{F}^{2}+{\left\|C\right\|}_{F}^{2}\right)\;+\;\nu \sum_{k=1}^{K}\;\sum_{t=1}^{L}{\left(c_{t,k}-\beta_{k}S_{k}(t-1)I_{k}(t-1)\right)}^{2}\;\text{s. t.}\;A\geq 0,B\geq 0,C\geq 0,\;\beta \geq 0,\gamma \geq 0,s\geq 0,i\geq 0,\;S_{k}(t)=S_{k}(t-1)-\beta_{k}S_{k}(t-1)I_{k}(t-1),\;I_{k}(t)=I_{k}(t-1)+\beta_{k}S_{k}(t-1)I_{k}(t-1)-\gamma_{k}I_{k}(t-1),\;s_{k}=S_{k}(0),i_{k}=I_{k}(0).$$

The first term is the data fitting term. We fit a CPD model of rank-*K* with nonnegativity constraints on the factors. The second term is Frobenius norm regularization which is typically used to avoid overfitting and improve generalization of the model. We introduce a third term which regularizes each column of the factor matrix **C** according to Equation (2) i.e., we learn *K* different SIR models, each of which is fully described by parameters *S*_*k*_(0), *I*_*k*_(0), *R*_*k*_(0) and *β*_*k*_, *γ*_*k*_ according to Equations (1a), (1b), (1c). We aim at estimating these parameters such that each column of **C** follows the new infections curve of an SIR model. Vectors $\beta \in {\mathbb{R}}^{K}$ and $\gamma \in {\mathbb{R}}^{K}$ hold the *β* and *γ* parameters of each SIR model and vectors $s=\in {\mathbb{R}}^{K}$, $i\in {\mathbb{R}}^{K}$ hold the initial conditions of each SIR model. Note that the recovered cases curve can be safely removed because it does not affect the optimization cost and that the parameter *N*_*k*_ corresponding to the total population has been absorbed in the vector *β*.

### Prediction

After the convergence of the above optimization algorithm, we have some estimates of $\hat{A}$, $\hat{B}$, $\hat{C}$ and parameters {*β*_1_, ⋯ , *β*_*K*_}, {*γ*_1_, ⋯ , *γ*_*K*_}, {*s*_1_, ⋯ , *s*_*K*_}, {*i*_1_, ⋯ , *i*_*K*_} where the pair of *β*_*k*_ and *γ*_*k*_ describes the epidemic transmission of the *k*th component in the time factor matrix and *s*_*k*_, *i*_*k*_ the initial values of the subpopulations. Using Equations (1a), (1b) and (2) we can predict “future” values for the *k*th column of **C**. We repeat the same procedure for all columns of **C** such that we can predict the entire “future” rows. Let $\hat{C}(t,:)\in {\mathbb{R}}^{K}$ be the prediction of the temporal information at a future time point *t* using estimates $\hat{\beta }$, $\hat{\gamma }$, $\hat{s}$, $\hat{i}$. Since **A** and **B** do not depend on *t*, the prediction of all signals in the tensor at time *t* is given by

(5) $$\hat{\underline{X}}(:,:,t)=\hat{A}\text{diag}(\hat{C}(t,:){\hat{B}}^{T}.$$

Adding latent temporal regularization through an SIR model offers significant advantages compared to having separate SIR models for each location. Our model can capture correlations between different locations and signals through their latent representations and therefore improve the prediction accuracy. Additionally, it enables expressing the evolution of a signal as weighted sum of *K* separate SIR models e.g., the prediction of the *n*th signal for the *m*th location for time point *t*, is given by

(6) $$\hat{\underline{X}}(m,n,t)=\sum_{k=1}^{K}\;{\hat{a}}_{m,k}{\hat{b}}_{n,k}{\hat{\beta }}_{k}S_{k}(t-1)I_{k}(t-1),$$

which makes it much more flexible and expressive.

### Optimization

The optimization problem (4) is a nonconvex and very challenging optimization problem. To update factor matrices **A**, **B**, **C** we rely on alternating optimization. Note that by fixing all variables except for **A**, the resulting subproblem w.r.t. **A**, is a nonnegative least squares problem, which is convex. Similarly for the factor matrices **B** and **C**. We choose to solve each factor matrix subproblem via the Alternating Direction Method of Multipliers (ADMM) (Gabay and Mercier 1976), which is a very efficient algorithm that has been successfully applied to nonnegative tensor factorization problems (Huang, Sidiropoulos, and Liavas 2016).

Let us first consider the subproblem w.r.t. **A**. Assume that at the *ℓ*th iteration, we have some estimates of all the variables available. Fixing all variables except for **A**, we have

(7) $$\underset{A\geq 0}{\text{min}}\;{\left\|{\underline{X}}^{\left(1\right)}-\Phi_{A}^{\left(l\right)}A^{T}\right\|}_{F}^{2}+\mu {\left\|A\right\|}_{F}^{2}$$

where $\Phi_{A}^{\left(l\right)}=C^{\left(l\right)}⊙B^{\left(l\right)}$. The ADMM updates for optimization problem (7) are the following

(8a) $$\hat{A}=\text{arg}\;\text{min}{\left\|{\underline{X}}^{\left(1\right)}-\Phi_{A}^{\left(l\right)}\hat{A}\right\|}_{F}^{2}+\mu {\left\|\hat{A}\right\|}_{F}^{2}+\rho {\left\|A-{\hat{A}}^{T}+A_{d}\right\|}_{F}^{2},$$

(8b) $$A=\underset{A\geq 0}{\text{arg}\;\text{min}}\rho {\left\|A-{\hat{A}}^{T}+A_{d}\right\|}_{F}^{2},$$

(8c) $$A_{d}=A_{d}+A-{\hat{A}}^{T}.$$

Equation (8a) is a least squares problem. Because ADMM is an iterative algorithm and the update is performed multiple times, we save computations by caching (Huang, Sidiropoulos, and Liavas 2016). Equation (8b) is a simple elementwise nonnegative projection operator and Equation (8c) is the dual variable update. The updates for **B** are similar.


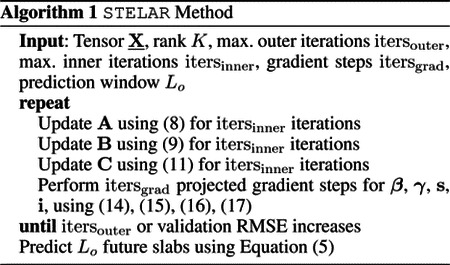

(9a) $$\hat{B}=\text{arg}\;\text{min}{\left\|{\underline{X}}^{\left(2\right)}-\Phi_{B}^{\left(l\right)}\hat{B}\right\|}_{F}^{2}+\mu {\left\|\hat{B}\right\|}_{F}^{2}+\rho {\left\|B-{\hat{B}}^{T}+B_{d}\right\|}_{F}^{2},$$

(9b) $$B=\underset{B>0}{\text{arg}\;\text{min}}\rho {\left\|B-{\hat{B}}^{T}+B_{d}\right\|}_{F}^{2},$$

(9c) $$B_{d}=B_{d}+B-{\hat{B}}^{T}.$$

where $\Phi_{B}^{\left(l\right)}=C^{\left(l\right)}⊙A^{\left(l\right)}$. Now let us consider the update of **C**. The related optimization problem takes the form of

(10) $$\underset{C>0}{\text{min}}\;{\left\|{\underline{X}}^{\left(3\right)}-\Phi_{C}^{\left(l\right)}C^{T}\right\|}_{F}^{2}+\mu {\left\|C\right\|}_{F}^{2}+\nu {\left\|C-{\bar{C}}^{\left(l\right)}\right\|}_{F}^{2},$$

where $\Phi_{C}^{\left(l\right)}=B^{\left(l\right)}⊙A^{\left(l\right)}$, ${\bar{C}}^{\left(l\right)}=\left(P^{\left(l\right)}⊛Q^{\left(l\right)}\right)\text{diag}\left(\beta^{\left(l\right)}\right)$ and we define $P^{\left(l\right)}(t,k):=S_{k}(t-1)$ and $Q^{\left(l\right)}(t,k):=I_{k}(t-1)$. Optimization problem (10) is also a nonnegative least squares problem. Therefore the updates for **C** are

(11a) $$\hat{C}=\text{arg}\;\text{min}{\left\|{\underline{X}}^{\left(3\right)}-\Phi_{C}^{\left(l\right)}\hat{C}\right\|}_{2}^{2}+\mu {\left\|\hat{C}\right\|}_{F}^{2}+\nu {\left\|C-{\bar{C}}^{\left(l\right)}\right\|}_{F}^{2}+\rho {\left\|C-{\hat{C}}^{T}+C_{d}\right\|}_{F}^{2}$$

(11b) $$C=\underset{C\geq 0}{\text{arg}\;\text{min}}\rho {\left\|C-{\hat{C}}^{T}+C_{d}\right\|}_{F}^{2}$$

(11c) $$C_{d}=C_{d}+C-{\hat{C}}^{T}$$

We observed that running a few ADMM inner iterations (~ 10) for each factor suffices for the algorithm to produce satisfactory results. By fixing the factor matrices, we have

(12) $$\underset{\beta ,\gamma ,s,i}{\text{min}}\sum_{k=1}^{K}\;\sum_{t=1}^{L}\;{\left(c_{t,k}-\beta_{k}S_{k}(t-1)I_{k}(t-1)\right)}^{2}\;\text{s. t.}\;\beta \geq 0,\gamma \geq 0,s\geq 0,i\geq 0,\;S_{k}(t)=S_{k}(t-1)-\beta_{k}S_{k}(t-1)I_{k}(t-1),\;I_{k}(t)=I_{k}(t-1)+\beta_{k}S_{k}(t-1)I_{k}(t-1)-\gamma_{k}I_{k}(t-1),\;s_{k}=S_{k}(0),i_{k}=I_{k}(0).$$

Both *S*_*k*_(*t*) and *I*_*k*_(*t*) are functions of *β* and *γ*, and are calculated in a recursive manner. Therefore the optimization problem w.r.t. *β* and *γ* is nonconvex. Optimization problem (12) corresponds to *K* independent one-dimensional curve fitting problems and since there are only 4 optimization variables for each problem, we can use off-the-shelf curve fitting methods. Alternatively, we can perform a few projected gradient descent steps. Focusing on the *k*th subproblem we have

(13) $$f\left(\beta_{k}\right)=\nu \sum_{t=1}^{L}\;{\left(c_{t,k}-\beta_{k}S_{k}(t-1)I_{k}(t-1)\right)}^{2}.$$

The derivative of *f*(*β*_*k*_) w.r.t. *β*_*k*_ is

(14) $$\frac{\partial f}{\partial \beta_{k}}=-2\nu \sum_{t=1}^{L}\;\left(c_{t,k}-\beta_{k}S_{k}(t-1)I_{k}(t-1)\right)\times \left(S_{k}(t-1)I_{k}(t-1)+\beta_{k}\frac{\partial S_{k}(t-1)}{\partial \beta_{k}}I_{k}(t-1)\;+\beta_{k}S_{t-1,k}\frac{\partial I_{t-1,k}}{\partial \beta_{k}}\;\right).$$

Note that both *S*_*k*_(*t*) and *I*_*k*_(*t*) are recursive functions. Thus, their respective derivatives w.r.t. *β* are computed recursively in *L* steps. Similarly, for *γ*_*k*_ we have

(15) $$\frac{\partial f}{\partial \gamma_{k}}=-2\nu \sum_{t=1}^{L}\;\left(c_{t,k}-\beta_{k}S_{k}(t-1)I_{k}(t-1)\right)\times \left(\beta_{k}\frac{\partial S_{t-1}}{\partial \gamma }I_{t-1}+\beta_{k}S_{k}(t-1)\frac{\partial I_{k}(t-1)}{\partial \gamma }\right).$$

Finally for **s**,**i**

(16) $$\frac{\partial f}{\partial s_{1}}=-2\nu \sum_{t=1}^{L}\;\left(c_{t,k}-\beta_{k}S_{k}(t-1)I_{k}(t-1)\right)\times \left(\beta_{k}\frac{\partial S_{k}(t-1)}{\partial s_{1}}I_{k}(t-1)+\beta_{k}S_{k}(t-1)\frac{\partial I_{k}(t-1)}{\partial s_{1}}\right),$$

(17) $$\frac{\partial f}{\partial i_{1}}=-2\nu \sum_{t=1}^{L}\;\left(c_{t,k}-\beta_{k}S_{k}(t-1)I_{k}(t-1)\right)\times \left(\beta_{k}\frac{\partial S_{k}(t-1)}{\partial i_{1}}I_{k}(t-1)+\beta_{k}S_{k}(t-1)\frac{\partial I_{k}(t-1)}{\partial i_{1}}\right).$$

The overall procedure is summarized in Algorithm 1.

## Experiments

### Dataset and Baselines

We use US county-level data from the Johns Hopkins University (JHU) (Dong, Du, and Gardner 2020) and a large IQVIA patient claims dataset, which can be publicly accessible upon request. JHU data includes the number of active cases, confirmed cases and deaths for different counties in the US. The total number of counties was 133. We use the reported active cases to compute the daily new infections. The claims dataset was created from 582,2748 claims from 732,269 COVID-19 patients from 03-24-2020 to 06-26-2020 (95 days). It contains the daily counts of 12 International Classification of Diseases ICD-10 codes observed in each county and the Current Procedural Terminology (CPT) codes related to hospitalization and utilization of intensive care unit (ICU). The size of the constructed tensor is 133 × 15 × 95. Using this data, we also construct a tensor which includes the aggregated data for 5 states, New York, New Jersey, Massachusetts, Connecticut, Pennsylvania and is of size 5 × 15 × 95.

We perform two different experiments. Initially we use 85 days for training and validation and use the remaining *L*_*o*_ = 10 as the test set. In the second experiment we use 80 days for training and validation and *L*_*o*_ = 15 days for test. We compare our method against the following baselines:

1. **Mean.** We use the mean of the last 5 days of the training set as our prediction.
2. **SIR.** The susceptible-infected-removed model.
3. **SEIR.** The susceptible-exposed-infected-removed epidemiological model.
4. **LSTM (w/o feat.)** LSTM model without additional features. We use one type of time series as our input.
5. **LSTM (w feat.)** LSTM with additional features. We use 15 different time series as input.
6. **STAN** (Gao et al. 2020) A recently proposed GNN with attention mechanism.
7. **STELAR** (*ν* = 0) The proposed method without regularization (two-step approach).
  We use the Root Mean Square Error (RMSE) and Mean Absolute Error (MAE) as our evaluation metrics.

### Results

Table 2 shows the county-level results for 10 and 15 day prediction for daily new infections and hospitalized patients. For new infections and *L*_*o*_ = 10, our method achieves 18% lower RMSE and 9% lower MAE compared to the best performing baselines which are the SIR and STAN model respectively. When *L*_*o*_ = 15 our method achieves 10% lower RMSE and the same MAE compared to the STAN model. For hospitalized patients and *L*_*o*_ = 10, our method achieves 21% lower RMSE and 12% lower MAE compared to the best performing baseline which is STAN. When *L*_*o*_ = 15 our method achieves 12% lower RMSE and 25% lower MAE compared to the best baseline. For state-level prediction of new infections, the best performing model is STAN but for hospitalized patients our method again outperforms all the baselines. Note that in all cases except one, joint optimization and SIR model fitting always improves the performance of our method compared to the two-step procedure.

**Figure 2:**
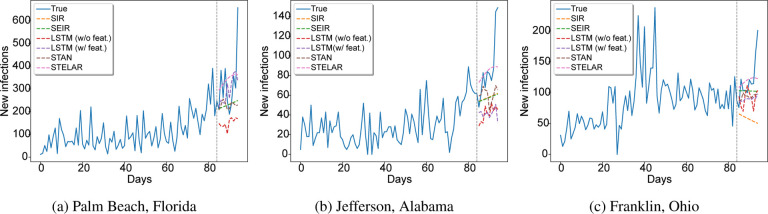
10 days county-level prediction for new infections.

Figure 2 shows some examples of county-level prediction. Figure 2a shows simple case where one can observe an increasing pattern of the new infections. Almost all models are able to capture this trend except for the LSTM (w/feat.) model. Figure 2b shows a more challenging scenario where it is not obvious if the curve will continue increasing but our method makes an accurate prediction, and is better than the baselines. Finally in Figure 2c we observe an example where a peak was already observed in the past and therefore SIR and SEIR models fail. On the other hand, our method is again able to make reasonable predictions.

**Figure 3:**
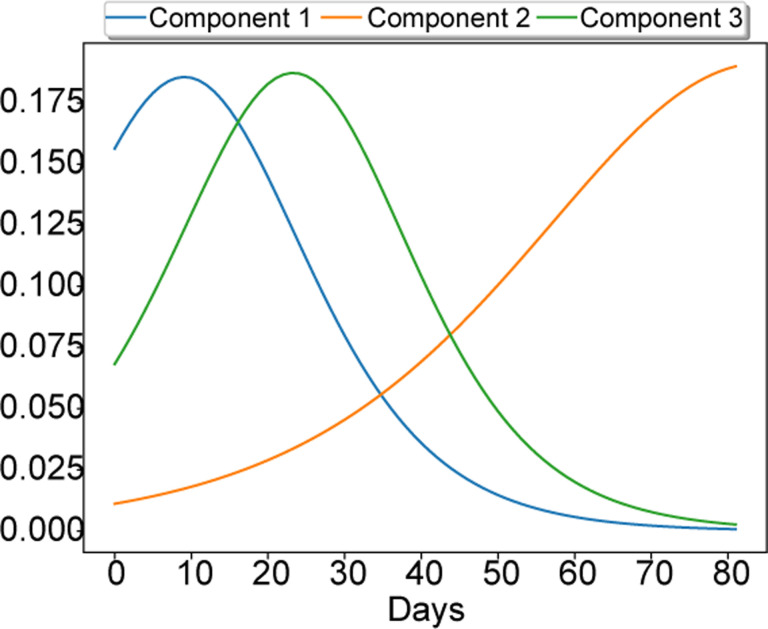
The strongest 3 temporal components of a rank-30 STELAR model.

Finally, we demonstrate the ability of our model to produce interpretable results. We train our model on county-level data using *K* = 30. After the algorithm converges we normalize each factor matrix such that each column has unit norm and absorb the scaling to a vector *w*. Using this vector, we extract the 3 rank-1 components with the highest weights i.e., by selecting the corresponding columns of **A**, **B**, **C**. Figure 3 depicts the 3 temporal profiles (columns of factor **C**) that corresponds to the selected rank-1 components. We observe that the different curves depict different stages of the disease transmission. For example, the 1st component has captured an initial wave of the disease, the 2nd has captured a more recent increasing trend and the 3rd component is very similar to the first but shifted to the right. To gain some intuition regarding the results, we sort each column of factor matrices **A** (counties) and **B** (signals) and present the top 10 counties and top 5 signals that contribute to each one of the 3 strongest components in Tables 4, 5. We can clearly see that the counties which correspond to the early wave (1st component) are from the states of New York and New Jersey as we would expect. Also the 1st component is mostly associated with new infections, hospitalized patients and ICU. On the other hand, the 3rd component which is very similar to the 1st is associated with hospitalized patients and ICU but not with new infections. Some counties appear in both the 1st and 3rd component which means that hospitalized patients and ICU cases started to increase (or getting reported) slightly after the new infections were reported. Finally, the second component depicts a later increase to the number of infections and hospitalized patients for some counties.

## Conclusion

In this paper, we propose STELAR a data efficient and interpretable method based on constrained nonnegative tensor factorization. Unlike standard tensor factorization methods, our method enables long-term prediction of future slabs by incorporating latent epidemiological regularization. We demonstrated the ability of our method to make accurate predictions on real county- and state-level COVID-19 data. Our method achieves 18% and 10% lower RMSE compared to the best baseline, when predicting county-level daily new infections for 10 and 15 days-ahead respectively and 21% and 12% lower RMSE when predicting county-level hospitalized patients.

## Figures and Tables

**Table 1:** Notation

Notation	Description
$\underline{X}\in {\mathbb{R}}^{M\times N\times L}$	spatio-temporal tensor
$A\in {\mathbb{R}}^{M\times K}$	location factor matrix
$B\in {\mathbb{R}}^{N\times K}$	signal factor matrix
$C\in {\mathbb{R}}^{L\times K}$	temporal factor matrix
${\underline{X}}^{\left(i\right)}$	mode-i unfolding of $\underline{X}$
*β*; *γ*;	contact rate; recovery rate
*K*	# of components
*M*	# of locations
*N*	# of signals
*L*	# of time points
*T*	transpose
∘	outer product
⊗	Kronecker product
⊙	Khatri-Rao product
⊛	Hadamard product
‖·‖_*F*_	Frobenius norm
diag(x)	diagonal matrix

**Table 2:** County-level prediction for new infections (left) and hospitalized patients (right).

	*L*_*o*_ = 10		*L*_*o*_ = 15				*L*_*o*_ = 10		*L*_*o*_ = 15	
Model	RMSE	MAE	RMSE	MAE		Model	RMSE	MAE	RMSE	MAE
Mean	304.1	122.0	269.5	108.5		Mean	125.0	77.0	123.3	77.1
SIR	156.2	62.2	159.1	63.6		SIR	46.5	27.2	48.7	27.7
SEIR	177.1	72.9	163.2	69.7		SEIR	39.1	23.9	41.1	25.7
LSTM (w/o feat.)	203.6	77.1	191.0	81.7		LSTM (w/o feat.)	45.6	23.6	54.8	31.2
LSTM (w/ feat.)	162.3	68.2	187.6	78.3		LSTM (w/ feat.)	42.5	23.3	47.5	26.8
STAN	164.2	61.1	152.6	61.8		STAN	30.6	17.3	42.8	24.2
STELAR (*v* = 0)	149.2	61.5	152.8	66.9		STELAR (*v* = 0)	28.6	16.6	46.8	21.1
STELAR	**127.5**	**55.6**	**136.1**	**61.7**		STELAR	**24.0**	**15.1**	**36.0**	**18.0**

**Table 3:** State-level prediction for new infections (left) and hospitalized patients (right).

	*L*_*o*_ = 10		*L*_*o*_ = 15				*L*_*o*_ = 10		*L*_*o*_ = 15	
Model	RMSE	MAE	RMSE	MAE		Model	RMSE	MAE	RMSE	MAE
Mean	309.0	258.7	325.8	273.1		Mean	685.5	553.9	729.2	586.0
SIR	186.1	133.8	186.9	134.5		SIR	343.4	252.4	367.8	266.0
SEIR	162.4	127.0	162.6	130.2		SEIR	109.0	97.6	192.5	154.3
LSTM (w/o feat.)	187.5	138.1	419.7	356.0		LSTM (w/o feat.)	280.9	187.5	416.1	308.7
LSTM (w/ feat.)	197.9	151.6	359.2	286.5		LSTM (w/ feat.)	295.3	182.3	276.0	208.5
STAN	**74.1**	**60.1**	**100.5**	79.6		STAN	100.3	73.6	177.7	144.7
STELAR (*v* = 0)	140.8	104.0	127.8	95.0		STELAR (*v* = 0)	118.3	84.4	126.6	**75.2**
STELAR	117.8	89.8	107.3	**79.4**		STELAR	**56.8**	**43.9**	**113.6**	83.8

**Table 4:** Counties that contribute more to each of the strongest 3 rank-1 components of a rank-30 STELAR model.

Component 1	Component 2	Component 3
New York (NY)	L.A (CA)	Nassau (NY)
Westchester (NY)	Cook (IL)	L.A (CA)
Nassau (NY)	Milwaukee (WI)	Essex (NJ)
Bergen (NJ)	Fairfax (VA)	Wayne (MI)
Miami-Dade (FL)	Hennepin (MN)	Oakland (MI)
Hudson (NJ)	Montg. (MD)	Middlesex (NJ)
Union (NJ)	P. George’s (MD)	New York (NY)
Phila. (PA)	Dallas (TX)	Phila. (PA)
Passaic (NJ)	Orange (CA)	Cook (IL)
Essex (NJ)	Harris (TX)	Bergen (NJ)

**Table 5:** Signals that contribute more to each of the strongest 3 rank-1 components of a rank-30 STELAR model. Definitions of ICD-10 codes: J96–Respiratory failure, not elsewhere classified; N17–Acute kidney failure; R05–Cough. R06–Abnormalities of breathing; R09–Other symptoms and signs involving the circulatory and respiratory system.

Component 1	Component 2	Component 3
New infections	New infections	Hosp. patients
Hosp. patients	Hosp. patients	ICU patients
ICU patients	ICU patients	J96
J96	J96	N17
R09	R05	R06
